# The politics of health system quality: how to ignite demand

**DOI:** 10.1136/bmj-2023-076792

**Published:** 2023-12-09

**Authors:** Kevin Croke, Gagan K Thapa, Amit Aryal, Sudip Pokhrel, Margaret E Kruk

**Affiliations:** 1 Harvard T H Chan School of Public Health, Boston, MA, USA; 2 Federal Parliament of Nepal, Baneshwor, Kathmandu, Nepal; 3 Swiss TPH and University of Basel, Basel, Switzerland; 4 Office of Member of Parliament Gagan K Thapa, Naxal, Kathmandu, Nepal

## Abstract

**Kevin Croke and colleagues** consider how demand for quality health systems can be made a political and public priority to drive change in low and middle income countries

The root causes of gaps in quality of care in the health systems of low and middle income country generate considerable debate, and opinions differ about how to tackle these gaps. The debate is illustrated by three important reports published in 2018.^1^ ^2^ ^3^ The consensus view of major global health institutions is well captured by the 2018 report from the World Health Organization, World Bank, and Organisation for Economic Cooperation and Development, which emphasised technical strategies to improve system quality such as changes to payment systems, adoption of new technologies, and scale-up of facility level quality improvement interventions. This approach is consistent with most published evidence in the quality improvement field, which explicitly or implicitly takes the same approach. The Lancet Global Health Commission for High Quality Health Systems, by contrast, argued that quality interventions focused on frontline services were effectively sticking plasters, unlikely to drive systemic change. Real progress would require fundamental, system level changes in medical education, service delivery models, measurement, and health system governance. This perspective drove the argument that “moving to a high quality health system … is primarily a political, not a technical, decision.”^1^

Both the Lancet commission and the 2018 report on health system quality from the National Academies of Medicine, Science and Engineering highlighted the importance of “igniting” or “activating” demand for quality.^2^ ^3^ But neither offered a detailed explanation of where that demand might come from and how it could be cultivated. Drawing on theory and evidence from political science and public policy studies, as well as recent experiences from Nepal, we argue that existing thinking about demand for health system quality in low and middle income countries is incomplete. Bottom-up demand, from the public, and top-down mandates, from reform oriented leaders, are unlikely to be effective in isolation, and need to be supported by a network of quality focused researchers, activists, and government officials.

## Health system improvements require political action

Other fundamental health system reforms have largely been achieved through political processes. For example, case studies of breakthroughs in universal health coverage highlight the importance of political change—such as through constitutional reform, democratisation, or political mobilisation of previously marginalised groups—to the process.^4^ Yet the politics of quality focused reforms may be more challenging. Whereas universal health coverage has had broad political appeal, and has even been used as a campaign issue in some settings,^5^ ^6^ politicians rarely make health system quality a feature of their campaigns.

The idea of igniting population demand for health system quality is rooted in the assumption that political leaders seek to deliver public services, such as healthcare, to win voter approval.^7^ However, recent social science findings complicate this picture. Studies have shown that voters are surprisingly unresponsive, in aggregate, to new information about the performance of public officials.^8^ They seem more responsive to their experiences of public services than to information alone, without personal experience, but even here citizens seem most responsive to governmental actions that are visible and salient. Meta-analysis of experimental evidence shows that voters reward incumbent politicians for direct, clearly attributable actions,^9^ such as provision of cash transfers, but even in this case the effects are modest and variable. With this in mind, aspects of healthcare not easily defined or judged by patients or policy makers, such as competence of providers, are unlikely to shift opinion or motivate action, especially since for many users, interactions with the health system are often sporadic and irregular. Recent work on the politics of maternal health service quality in Ghana has documented how these dynamics may play out in practice.^10^

This poses a stark challenge for the project of igniting demand for quality. Construction of new hospitals is highly visible but often weakly correlated with quality of care, and most actions to improve health system quality are less tangible. Even in the best scenario, health is only one of many issues that politicians are responsible for, and quality is only one element of health.

Outside of electoral processes, bottom-up pressure can also emerge through social movements. However, when such movements have emerged in the health sector, they have typically focused on access, equity, or treatment for specific conditions, rather than broader health system quality.^11^ Demand for health system quality from citizens is unlikely to emerge on its own, without other supportive institutions and processes.

Demand for health system quality could also come from political leaders. While the public is rarely mobilised around health system quality, could it be enough to convince a president, ministers, or a group of parliamentarians, who could then use executive or legislative authority to push through needed reforms? This is the basis of “political will” theories of health system change. Reformist ministers can take positive steps, such as developing national quality strategies and creating quality focused units in ministries of health. They may also use other top-down instruments, such as changes to payment systems, to spur quality improvement. Yet here, as before, recent social science findings provide grounds for scepticism.

Health services have been described as transaction intensive but discretionary activities^12^—that is, health system quality, is determined by so many actions, taken so frequently, by so many people who have professional discretion to make autonomous decisions, that command and control attempts to control their behaviour are unlikely to succeed. Attempts to incentivise quality through performance targets have similarly had limited success. Clinical staff have jobs that cannot be reduced to algorithms; as a result, top-down diktats or application of performance indicators can quickly devolve into disputes about how to accurately measure quality. Perhaps for these reasons, there does not seem to be strong evidence that pay-for-performance programmes lead to major improvements in quality of care in low and middle income countries.^13^ ^14^ Although top-down efforts, including changes to provider payment or evaluation and measurement systems, can help when they are implemented with strong support from high level health system leaders, they can also be derailed when governments change and ministers of health leave office. Like bottom-up demand, high level political will may be a necessary element for health system transformation, but it is likely insufficient by itself.

## Are there other drivers of demand for quality?

So where is sustained demand for quality likely to come from, if not exclusively from the public or from politicians? In public policy studies, specialised policy domains (“policy subsystems”) are often seen as dominated by “iron triangles,” comprised of public sector bureaucracy, interest groups representing private sector interests, and legislators. These self-reinforcing triangles can limit public input and help commercial interests to capture policy. This concept often describes policy making on issues with substantial technical content (such as quality of care).

These inward focused triangles are not inevitable, however. Political scientists have also identified conditions under which similar groups can coalesce into public oriented, outward facing “issue networks.”^15^ Like iron triangles, these are loosely affiliated groups of specialists who work on a single policy topic, although these networks tend to be more fluid, less dominated by commercial interests, and more inclusive of academics, non-governmental organisations, journalists, and activists. They are also more likely to focus on research, expertise, and knowledge sharing.

## Role of issue networks

Effective, sustained demand for health system quality is more likely to come from the emergence of a thriving issue network focused on quality than from purely top-down or bottom-up approaches. Issue networks can provide an essential middle layer that connects top-down and bottom-up approaches, harnessing the bottom-up voices and pressures for accountability and uniting them with top-down technical expertise and political will. While issue networks often lack financial resources or direct political influence, they can exercise power indirectly by shaping public and experts’ understanding of issues through their technical expertise.

At the same time, their open structure allows them to channel diverse input and feed it into policy processes. For example, quality focused researchers can generate new measurement approaches, document quality gaps in a country’s health system, and test potential solutions, while journalists, non-governmental organisations, and activists can publicise these issues and raise them to government and public attention. Issue networks can forge expert consensus around potential policy solutions, so that when a political window of opportunity opens, experts have a policy response ready when politicians ask for options.^16^

However, effective networks do not emerge automatically. For neglected issues such as health system quality, it is challenging to foster a dense ecology of influential people and organisations. In high income countries the literature has largely focused on the characteristics of the target issue, the power, resources, and cohesion of the network, and the surrounding political context. More recently, literature on the effectiveness of health focused issue networks in low and middle income countries has also emerged. One finding is that issue networks are most effective when they capture the idealism of civil society but also create direct links with government. For example, issue networks were instrumental in the push for universal health coverage in both Thailand and Brazil.^17^ In both cases they were rooted in professional movements of socially minded physicians, who established non-governmental organisations, conducted research and advocacy, developed links with international organisations, and opened dialogue with politicians. Critically, some members of these movements also took up positions in government, giving them resources to put in place systemic change.

Another factor is the extent to which institutions comprising the network have sustained (ie, not project based) funding under local governance and ownership. For example, the issue network focused on malaria in Tanzania has been effective because of long term investment in institutionalisation, sustainable resourcing, and organisational autonomy through creation of an independent trust, governed by a board with ministry of health, academic, and international participation.^18^

A final factor relates to whether network members take an explicitly political, coalition building approach, rather than remaining narrowly technical and academic. One analysis found that cross-national global health issue networks were most effective if they constructed a compelling framing of the issue, and if they effectively built a political coalition that included actors outside the health sector.^19^ While there is no single recipe for the success of issue networks, these factors increase the likelihood of successful network development.

Issue networks by themselves may also not be sufficient to produce meaningful change unless they are deliberately and institutionally linked to health system governance processes. In Nepal, researchers, policy makers, and activists have been trying to place health quality on the political agenda.^20^ ^21^ However this nascent network has not been institutionalised or linked to the policy process. For example, politicians have lacked concrete platforms such as parliamentary committees or caucuses, as well as policy dialogue led by academics or non-governmental organisations, to learn about health system quality. They have often been left out of health policy discussions or given information that is full of jargon and difficult to comprehend. As a result, when civil society, journalists, and even citizens have called attention to concerns about health system quality (such as drug shortages in remote health facilities or patients dying because of negligence), politicians have focused on cosmetic solutions rather than delving into the systemic causes. When they have attempted to take action on quality, they have defaulted to hospital construction campaigns rather than considering whether quality services are being delivered in existing facilities.^22^ ^23^ Similarly, Nepal has experimented with tools such as social audits and community scorecards that could have allowed citizens and policy makers to define and judge the less tangible aspects of health system quality. However, these tools were implemented as isolated initiatives detached from political processes^24^ and therefore failed to catalyse action on quality.

Successful issue networks also often have an international dimension. While local conditions are the main drivers of network success or failure, research has shown how advocates, when blocked by local political realities, can use their participation in international networks to access resources and information, and to bring new issue framings and norms to their context.^25^ One such effort, formed in response to the Lancet commission, is the Quality Evidence for Health System Transformation (QUEST) network (box 1). This, and similar efforts, seek to create a thriving ecosystem of researchers, policymakers, advocates, and citizens working together to build high quality health systems.

Box 1 **Quality Evidence for Health System Transformation Network (QUEST)**

QUEST is a global research consortium working on updated, more nimble measurement of quality, targeted testing of systemic reforms to improve quality, training junior researchers, and producing science of a global standard that is relevant locally. Its architecture requires partnership between researchers and policy makers in each country to promote policy action on the findings.

QUEST centres are situated in educational or public health institutions so that the work diffuses across the broader health research and policy sphere. For example, QUEST Ethiopia is jointly coordinated by the Ethiopia Public Health Institute, the Ministry of Health, and Addis Ababa University.

Researchers regularly share findings with ministries of health and local health actors: professional organisations, non-governmental and civil society organisations, multilaterals such as WHO and World Bank, foundations, and, in low income settings, donor countries. Seminars, workshops, reports, and publications aim to promote a common definition of quality and understanding of the evidence for improvement.

Generating strong demand for quality is a challenge for all health systems, at all levels of development. Demand does not emerge automatically. But quality focused reforms will be more sustainable if they can be supported by multidisciplinary local and global networks of specialists and advocates. Finding ways to strengthen and institutionalise such networks is an important area for future research and practice.

**Key messages**

- Generating greater demand for health system quality is a key element of quality improvement strategies in low and middle countries
- Yet most of these strategies have overlooked the political challenges that this entails
- Both top-down reforms by policy makers and bottom-up mobilisation from the public face systematic barriers
- Reformers should seek to cultivate issue networks focused on health system quality, as a complement to top-down and bottom-up approaches
- Experience from Nepal shows the additional value that can be gained from formally linking issue networks with existing political and administrative structures

## Notes

Contributors and sources: The authors have all collaborated on health system quality research through the QUEST network. MK co-led the 2018 Lancet Global Health Commission on High Quality Health Systems and currently leads the QUEST Network. KC studies the politics of health system reform and is a principal investigator for large scale quality improvement trials in Estonia and Kenya. GT is former minister of health of Nepal and a current member of parliament. AA and SP are health systems researchers and advisers to MPT. This article is based on a narrative review and synthesis of the existing literature on health system quality and on the public policy process, and on the authors’ experiences working on health system quality initiatives globally and in Nepal.

Provenance and peer review: Commissioned; externally peer reviewed.

This article is part of a collection proposed by the World Health Organization and the World Bank and commissioned by *The BMJ*. *The BMJ* peer reviewed, edited, and made the decision to publish these articles. Article handling fees are funded by the Bill and Melinda Gates Foundation. Jennifer Rasanathan, Juan Franco, and Emma Veitch edited this collection for *The BMJ*. Regina Kamoga was the patient editor.
